# Ablação por Cateter de Taquicardia Atrial Focal com Ativação Precoce Próxima ao Feixe de His, a Partir da Cúspide Aórtica não Coronária

**DOI:** 10.36660/abc.20180449

**Published:** 2021-01-27

**Authors:** Muhieddine Chokr, Lucas G. de Moura, Italo Bruno dos Santos Sousa, Cristiano Faria Pisani, Carina Abigail Hardy, Sissy Lara de Melo, Arnobio Dias da Ponte, Ieda Prata Costa, Ronaldo Vasconcelos Tavora, Luciana Sacilotto, Tan Chen Wu, Francisco Carlos da Costa Darrieux, Denise Tessariol Hachul, Vera Aiello, Mauricio Scanavacca

**Affiliations:** 1 Universidade de São Paulo Faculdade de Medicina Hospital das Clinicas São PauloSP Brasil Universidade de São Paulo Faculdade de Medicina Hospital das Clinicas Instituto do Coração, São Paulo, SP – Brasil; 2 Antonio Prudente Hospital FortalezaCE Brasil Antonio Prudente Hospital, Fortaleza, CE – Brasil

**Keywords:** Arritmias Cardíacas, Taquicardia Atrial, Ablação por Cateter/métodos, Fascículo Atrioventricular, Técnicas Eletrofisiológicas Cardíacas/métodos, Eletrocardiografia/métodos

## Abstract

**Fundamento:**

A ablação da taquicardia atrial (TA) com local de ativação mais precoce próxima ao feixe de His é um desafio, devido ao risco de bloqueio de AV completo por sua proximidade ao sistema de His-Purkinje (SHP). Uma alternativa para minimizar esse risco é posicionar o cateter na cúspide não coronária (CNC), que é anatomicamente contígua à região para-Hissiana.

**Objetivos:**

O objetivo deste estudo foi fazer uma revisão de literatura e avaliar as características eletrofisiológicas, a segurança e o índice de sucesso de aplicação de radiofrequência (RF) por cateter na CNC para o tratamento de TA para-Hissiana em uma série de casos.

**Métodos:**

Avaliamos retrospectivamente dez pacientes (Idade: 36±10 anos) que foram encaminhados para ablação de taquicardia paroxística supraventricular (TPSV) e haviam sido diagnosticados com TA focal para-Hissiana confirmada por manobras eletrofisiológicas clássicas. Para a análise estatística, um P valor d <0.05 foi considerado estatisticamente significativo.

**Resultados:**

A ativação atrial mais precoce na posição His foi de 28±12ms da onda P, e a CNC foi 3±2ms antes da posição His, sem evidência de potencial His em todos os pacientes. Foi aplicada RF à CNC (cateter de ponta de 4-mm; 30W, 55°C) e a taquicardia foi interrompida em 5±3s sem aumento no intervalo PR ou evidência de um ritmo juncional. Os testes eletrofisiológicos não induziram novamente a taquicardia em 9/10 pacientes. Não houve complicações em nenhum procedimento. Durante o período de acompanhamento de 30 ± 12 meses, nenhum paciente apresentou recorrência de taquicardia.

**Conclusão:**

O tratamento percutâneo de TA para-Hissiana por meio de CNC é uma estratégia segura e eficiente, tornando-se uma opção interessante para o tratamento de arritmia complexa. (Arq Bras Cardiol. 2021; 116(1):119-126)

## Introdução

As taquicardias atriais focais (TA) geralmente se originam de determinadas estruturas compostas de tecido atrial, tais como a *crista terminalis* , veias pulmonares, apêndices atriais e óstio do seio coronário. A ablação por cateter com radiofrequência (RF) foi estabelecida como o método preferencial para o tratamento dessas arritmias. Embora focos originados na região para-Hissiana sejam raros, eles são um desafio terapêutico devido à proximidade com o sistema de His-Purkinje (SHP). A tentativa de ablação via átrio direito poderia aumentar o risco de afetar o nó AV, SHP, e, portanto, causar o bloqueio atrioventricular (AV). Entretanto, o uso do acesso retroaórtico para explorar a cúspide não coronária (CNC), que é anatomicamente adjacente à região mencionada acima, é descrito como uma estratégia alternativa.^1^ A experiência com a eficácia e a segurança desse tipo de ablação continua limitada. Neste estudo, relatamos uma série de casos de taquicardia atrial para-Hissiana que foram mapeados e fizeram a ablação pela CNC. As características eletrofisiológicas e os resultados com essa abordagem foram analisados. Além disso, a anatomia da região e as estratégias procedimentais foram discutidas.

## Método

Foram analisados os prontuários de 10 pacientes (8 mulheres e 2 homens; média de idade 36±10 anos), de duas instituições brasileiras (Instituto do Coração/InCor, Faculdade de Medicina da Universidade de São Paulo. e Hospital Antônio Prudente, Fortaleza), submetidos a ablação por cateter entre janeiro de 2014 e março de 2017. O tratamento com drogas antiarrítmicas foi interrompido por pelo menos cinco meias-vidas antes do procedimento. Eles foram avaliados por exame físico, radiografia torácica, e ecocardiograma, e nenhum apresentou doença cardíaca estrutural.

Os pacientes foram submetidos a um estudo eletrofisiológico após jejum de 8 horas, sob monitoramento contínuo e nível de sedação controlado por um anestesista. Foi realizada punção tripla na veia femoral, e cateteres padrão (3) foram introduzidos no seio coronário (decapolar; 6F), região do feixe His (quadripolar, 7F), e base do ventrículo direito (quadripolar, 7F).

A estimulação atrial programada, ou ruptura atrial foi feita com um sistema de registro de eletrofisiologia (EP tracer, Holanda) para induzir a taquicardia em dois pacientes; e o aparecimento espontâneo de taquicardia foi observado em um paciente. O uso de isoproterenol (10-20mcg; infusão IV) foi necessário em sete pacientes. Em um caso, estava disponível um sistema de mapeamento eletroanatômico (Carto 3; Biosense).

O diagnóstico de TA foi confirmado utilizando-se as seguintes observações e manobras eletrofisiológicas: alterações no intervalo A-A antes de alterações no intervalo V-V, arrastamento ventricular durante a taquicardia com resposta tipo V-A-A-V, ou mesmo alterações no intervalo V-A durante a taquicardia (ausência de conexão V-A). Em todos os casos, observou-se a ativação atrial com menos de 50% do comprimento do ciclo de taquicardia, indicando um padrão de ativação focal.

Quando a ativação atrial mais precoce estava no septo atrial direito e era acompanhada de potencial His perceptível no local, a TA foi definida como para-Hissiana. Por último, a artéria femoral foi puncionada para permitir o mapeamento detalhado da região da válvula aórtica retrógrada.

**Figura 1:**
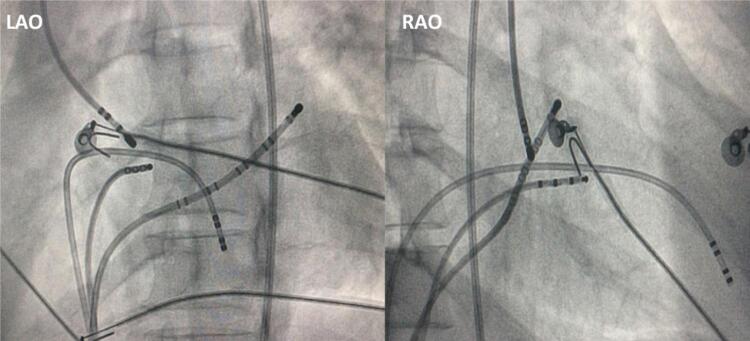
Posicionamento do cateter de ablação na CNC em projeção oblíqua direita e esquerda.

**Figura 2:**
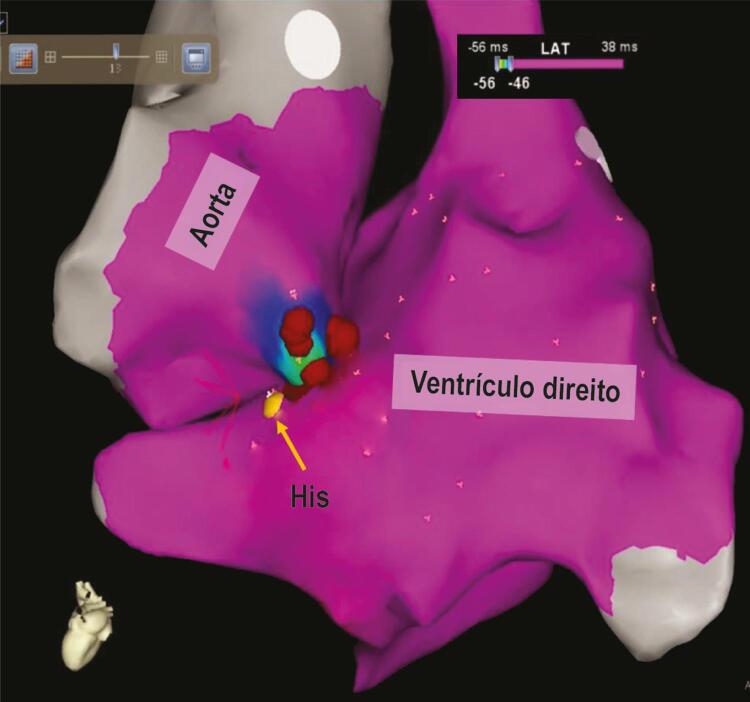
– Mapeamento eletroanatômico mostrando pontos de aplicação de RF na CNC. Observe o relacionamento íntimo da cúspide não coronária com a região para-Hissiana.

**Figura 3:**
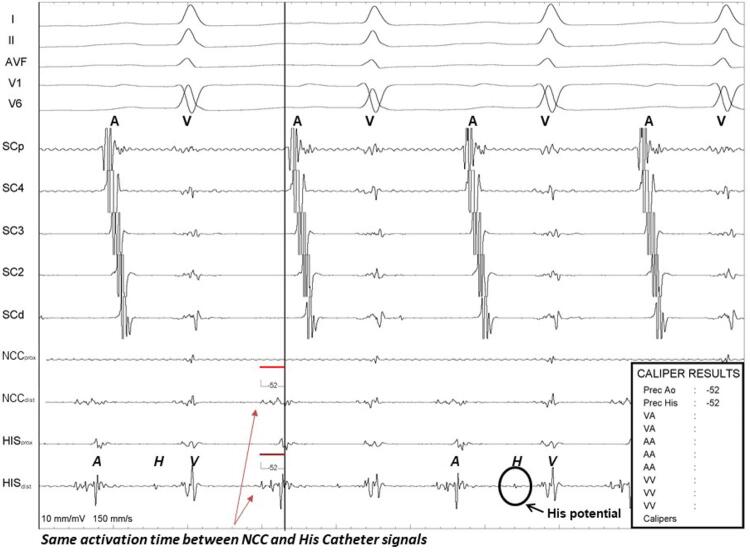
– Em sequência, de cima para baixo: derivações periféricas, seio coronário de eletrodos proximais e distais, e registro de cateteres próximos à região da válvula aórtica e do feixe de His. Tempo de ativação atrial local mais precoce semelhante é observado em relação ao aparecimento de uma onda P da região septal para-Hissiana e CNC, mas não se observou potencial His nesta última.

Um cateter terapêutico de ponta de 4-mm foi usado para aplicação de radiofrequência (RF) (30W/55ºC durante 60 segundos), usando incidências fluoroscópicas oblíquas direita e esquerda como referências para a localização anatômica ( Figura 1 ). Em um paciente, foi usado um sistema de mapeamento eletroanatômico ( Figura 2 ). Em todos os casos, o local da ativação mais precoce foi identificado pela CNC, em relação ao aparecimento da onda P periférica, semelhante ao detectado pelo cateter colocado no septo interatrial direito, mas com a vantagem da ausência do potencial de feixe de His no primeiro ( Figura 3 ). O sucesso do procedimento foi definido como sendo a cessação da taquicardia durante a aplicação de RF, e a não indução da taquicardia depois de várias tentativas de induzi-la por ruptura atrial, ou depois da infusão de isoproterenol.

### Análise estatística

Os dados contínuos são apresentados como média ± desvio padrão (DP), se forem normalmente distribuídos, e como mediana mais faixa interquartil, se não forem. Para dados categóricos, serão usadas contagens e porcentagens (%). O teste de Shapiro-Wilk foi usado para determinar a normalidade da distribuição. O teste U de Mann Whitney foi utilizado para comparar diferenças entre grupos para valores contínuos não paramétricos. Por último, o teste exato de Fisher foi aplicado para avaliação de dados categóricos em uma tabela de contingência 2x2. Para todos os testes, um P valor d <0,05 foi considerado estatisticamente significativo (bilateral). A análise estatística foi realizada com o software SPSS, versão 19.0 (SPSS Inc., Chicago, IL).

## Resultados

As características clínicas e eletrofisiológicas dos pacientes podem ser visualizadas na Tabela 1 e na Tabela 2 . Todas as variáveis contínuas, exceto a duração da onda P durante o ritmo do seio e a taquicardia, apresentaram distribuição normal ( Tabela 2 ).

**Tabela 1:** – As características clínicas dos pacientes avaliados

Paciente	Idade	Sexo	Doença cardíaca estrutural	Duração dos sintomas (meses)	AADs ineficazes	Ablação anterior mal sucedida
1	38	F	Nenhuma	≤12	0	Não
2	49	M	Nenhuma	12-24	2	Não
3	22	F	Nenhuma	12-24	1	Não
4	28	F	Nenhuma	12-24	0	Não
5	31	F	Nenhuma	≥48	3	Não
6	33	F	Nenhuma	≥48	1	Não
7	46	F	Nenhuma	≤12	1	Não
8	58	F	Nenhuma	12-24	2	Não
9	25	M	Nenhuma	≤12	0	Não
10	30	F	Nenhuma	≤12	1	Não

*AADs: drogas antiarrítmicas.*

**Tabela 2:** – Características eletrofisiológicas dos pacientes avaliados

Variável	Valor
Média de idade (anos)	36 ± 10*
Duração da onda p durante a taquicardia (ms)	93 ± 17^§^
Duração da onda p durante o ritmo do seio (ms)	112 ± 20^§^
Índice de sucesso da ablação	9/10 (90%)
Comprimento médio do ciclo de taquicardia (ms)	362±43*
Ativação atrial mais precoce registrada no cateter His (ms)	28±12*
Local da ativação mais precoce à região do feixe de His (ms)	3±2*
Tempo médio de fluoroscopia (minutos)	14 [10 - 18]
Tempo do início da ablação até a interrupção da taquicardia (segundos)	5 [2 - 8]
Uso de sistema de mapeamento 3D	1/10
Angiografia coronária	0/10
Complicações graves	0/10

*Os dados são apresentados como média + desvio padrão (DP). §O valor é exibido como média + faixa interquartil (IQR).

Nenhum dos pacientes já tinha passado por ablação por cateter. O comprimento médio do ciclo de taquicardia atrial foi 362±43 ms. Ativação atrial mais precoce registrada no cateter His foi 28±12 ms, em relação à onda P periférica. O tempo de ativação atrial local registrado pelo cateter na CNC foi 3±2 ms antes, comparado ao do cateter His.

Em todos os casos, o local inicial de ablação foi a CNC, com sucesso em 9 de 10 deles. O caso restante exigiu o mapeamento e a tentativa de ablação na região para-Hissiana com baixa potência (20w), que também não foi bem sucedido.

O tempo médio para a interrupção da taquicardia atrial depois da aplicação de RF foi 5 segundos. O ritmo juncional e um aumento no intervalo PR não foram observados durante a aplicação de RF em todos os casos. Todos os procedimentos foram bem tolerados e não houve complicações.

Durante um período de acompanhamento de 30 ± 12 meses, nenhum paciente teve recorrência de taquicardia atrial. Os pacientes permaneceram assintomáticos na avaliação clínica e com monitoramento por ECG Holter contínuo dinâmico.

**Figura 4:**
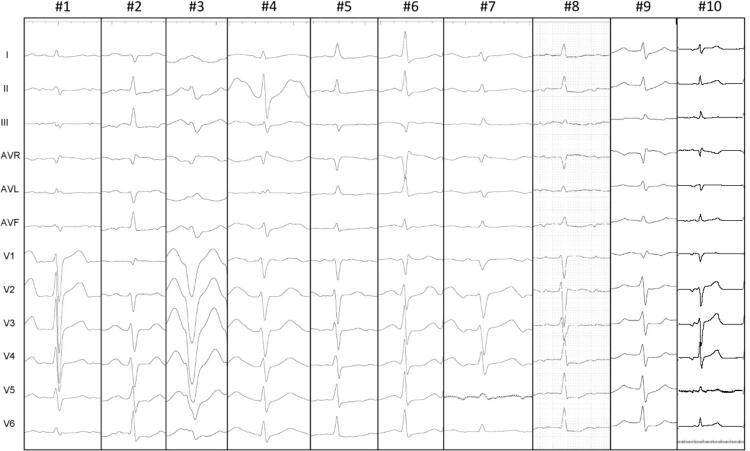
– Morfologia da onda P de todos os casos. Morfologia da onda P, demonstrando padrão bifásico ou trifásico em 6 de 10 pacientes em derivações inferiores.

Ao registrar o eletrocardiograma de superfície, observamos que a morfologia da onda P, demonstrando padrão bifásico ou trifásico em 6 de 10 pacientes em derivações inferiores, e teve duração significativamente mais curta, em comparação com o ritmo do seio em todos os casos, 93 ± 17 versus 112 ± 20 ms (p<0,05). ( Figura 4 ).

## Discussão

### Relações morfológicas

**Figura 5:**
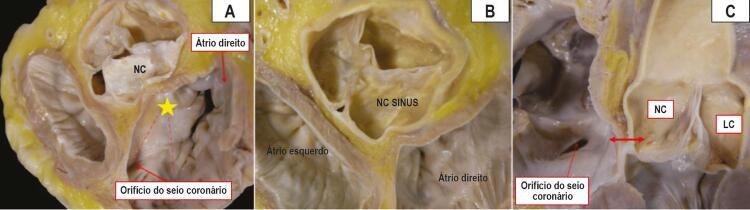
– Pode-se observar uma relação íntima entre o CNC e a região do feixe de His. A) Vista oblíqua de um corte de eixo curto na base do coração, mostrando o seio não coronário de Valsalva (NC) e os marcos do triângulo de Koch (linhas pontilhadas) e septo membranoso (estrela). B) Corte de eixo curto na base do coração mostrando as relações espaciais dos seios aórticos e o tecido adiposo presente entre a aorta e as paredes atriais. C) Seção longitudinal da raiz aórtica mostrando a curta distância entre a porção profunda do seio não coronário de Valsalva (CNC) e a área correspondente ao vértice do triângulo de Koch, localizado na região anterossuperior em relação ao orifício do seio coronário (seta com duas pontas).

A aorta ocupa uma posição central na base do coração, encaixada profundamente entre as junções atrioventriculares direita e esquerda. As relações espaciais dos seios de Valsalva demonstram, portanto, proximidade entre as paredes atriais e o tecido adiposo interposto entre eles na base do coração. Considerando um padrão de meia-lua da fixação dos componentes aórticos, é evidente que as relações topográficas variam de acordo com a profundidade dentro do seio. A Figura 5A e a Figura 5B mostram as relações anatômicas do seio aórtico não coronário em relação às estruturas do átrio direito. Especificamente, a parte mais profunda do seio aórtico não coronário (também chamado não adjacente) está intimamente relacionada ao componente atrioventricular do septo cardíaco. A Figura 5C mostra a parede do seio e os marcos da área juncional e nó atrioventricular

Dessa forma, a CNC passa a ser um alvo alternativo na estratégia terapêutica em que a falha na intervenção ocorre quando se tenta fazer a ablação nos dois lados do septo atrial, ou o risco de bloqueio atrioventricular resulta do registro de eletrograma do feixe de His próximo ao alvo da ablação.^2^

Do ponto de vista embriológico, células da crista neural contribuem para formar o septo aortopulmonar, coxim endocárdico no canal de saída, e isolamento do feixe de His do miocárdio ao seu redor. Resquícios dessas células na região perinodal pode justificar o substrato, que causa e mantém a arritmia.^3^ A CNC se origina do miocárdio atrial, enquanto a cúspide coronária esquerda se origina do miocárdio ventricular. Esse fato explica a frequência de arritmias atriais na NCC, e arritmias ventriculares nas cúspides direita e esquerda.^3^

A prevalência de taquicardias originárias da região perinodal é de 7 a 10% em séries diferentes, com várias séries e relatos de casos mostrando que taquicardias para-Hissianas podem ser tratadas adequadamente, com um índice de complicação baixo.^4^

A abordagem dessas taquicardias através da CNC reduz o risco de danos ao sistema de condução, garantindo uma estabilidade maior ao cateter durante a aplicação de RF, bem como um bom contato com o tecido. A explicação provável para a eficiência da ablação nesse local é o direcionamento das extensões atriais direitas na CNC, mais distante do sistema His Purkinje, que se localiza no endocárdio.^5^

Em relação a complicações, a aplicação de RF pode causar danos às válvulas cardíacas, embora essa complicação ainda não foi relatada até os limites de potência (30w) e temperatura (55°C) em várias séries.^6^ A angiografia coronária não foi realizada como rotina antes de se aplicar a RF porque nossa prática demonstra que a presença de um eletrograma com átrio maior que o ventrículo (razão A/V > 1), anatomicamente mais próximo a um cateter usado como referência no átrio direito, paralelo ao sistema de condução, marca um local seguro para a ablação. Em relação à técnica de mapeamento, observou-se uma razão maior ou igual a 1 entre as amplitudes dos eletrogramas atrial e ventricular em todos os pacientes do alvo de ablação. Essa característica eletrofisiológica tem grande valor, pois a inversão da relação A/V sugere que o limite da CNC está sendo ultrapassado e o cateter está apoiado na cúspide direita. Isso causa maior risco de lesão ao sistema de condução, servindo como assistência e referência anatômica quando a fluoroscopia é usada.^7^

Usamos o mapeamento eletroanatômico (MEA) apenas em um caso. O motivo disso é que a maioria de nossos pacientes foram tratados no sistema público de saúde, onde esse recurso não está disponível. Entretanto, em nossa amostra, conforme descrito por Toniolo et al.,^8^ foi possível alcançar altos índices de sucesso, apesar de não usar o recurso.

Por outro lado, há situações em que o MEA é fundamental. Recentemente, Bitaraes et al publicaram um caso de uma paciente grávida com uma TA refratária a tratamento farmacológico, em que a ablação por cateter foi realizada com sucesso pela cúspide aórtica não coronária com fluoroscopia zero, utilizando o MEA.^9^

Nossos achados estão em desacordo com os de Ouyang et al.,^10^ que observaram que uma onda P -/+ na derivação V1 associada a P+ em D1 e AVL sugerem origem na CNC. De acordo com esse autor, a relação entre a presença de uma onda P-/+ com sua parte mais proeminente sendo positiva e a origem no átrio esquerdo é um fato relevante. Recentemente, Madaffari et al.^11^ publicaram dados da morfologia da onda P, em que uma onda P estreita e bifásica (-/+) ou trifásica (+/-/+) nas derivações inferior e precordial identificam com confiança o grupo de TA que surge na região para-Hissiana. Em nosso estudo, identificamos que a morfologia da onda P, demonstrando padrão bifásico ou trifásico em 6 de 10 pacientes em derivações inferiores, e uma onda P significativamente mais curta em comparação com o ritmo do seio, era variável nas derivações precordiais.

Em nosso estudo, em dez casos, ocorreu apenas uma tentativa mal sucedida de realizar a ablação de taquicardia pela CNC, que também não teve sucesso pelo átrio direito. Presumimos que uma estratégia mais agressiva pelo lado direito do septo poderia ter resultado em danos ao sistema de condução e bloqueio atrioventricular, justificando a baixa potência de saída testada (20w). A taquicardia parou durante as aplicações, mas pode ser induzida novamente durante a infusão de isoproterenol. Um alvo mais profundo na região do septo poderia explicar a dificuldade de eliminar o substrato. Outra limitação é que o operador não explorou a região esquerda do septo nesse caso. Também não usamos cateter irrigado porque, em nossa opinião, a raiz aórtica é uma região de alto fluxo de sangue e, a menos que a aplicação da potência fosse limitada por cortes de alta temperatura repetidamente, a irrigação não deveria fazer uma diferença significativa. No acompanhamento clínico, o paciente estava assintomático, com o uso de betabloqueadores. Portanto, nenhuma nova tentativa de ablação foi realizada.

Recentemente, Lyan et al.,^12^ avaliaram estratégias diferentes de ablação por cateter de taquicardia atrial local originando próximo à região do feixe de His em 68 pacientes, e concluíram que o índice de sucesso agudo de ablação de TA para-Hissiana pela CNC foi mais alto do que a ablação no septo do AE e no septo do AD (p<0,05). Por esse motivo, eles argumentam que a CNC deve ser a primeira abordagem e a preferencial para essas taquicardias, independentemente do tempo de ativação local. Esse também e o posicionamento de Bohora et al.,^13^ Por outro lado, Madaffari et al.,^11^ propõem que a CNC é uma entre três abordagens possíveis para alcançar o sucesso, e que a escolha deve ser baseada no momento de ativação local.

Concordamos com Lyan et al.,^12^ e Bohora et al.,^13^ já que a abordagem de CNC parece ser a primeira escolha para realizar a ablação nesse cenário, com altos índices de sucesso.

## Conclusão

Este estudo confirma as observações prévias de que o mapeamento e a ablação por cateter de taquicardia atrial focal com ativação precoce próxima ao feixe de His, a partir da cúspide aórtica não coronária (CNC) é um procedimento eficiente e aparentemente seguro. Acreditamos que a exploração retroaórtica deva ser obrigatória nesse caso. Um eletrocardiograma de superfície pode sugerir o alvo adequado próximo à região do feixe de His, mas não em todos os casos. O conhecimento das relações da CNC com o sistema de condução é fundamental na ablação dessas taquicardias. Esses achados devem ser considerados na estratégia terapêutica dessa arritmia complexa.
