# Supplementary material for: Ablação por Cateter de Taquicardia Atrial Focal com Ativação Precoce Próxima ao Feixe de His, a Partir da Cúspide Aórtica não Coronária
Source: Arq Bras Cardiol. 2021 Jan 27;116(1):119–26. [Article in Portuguese] doi: 10.36660/abc.20180449 (PMC8159493; doi:10.36660/abc.20180449)
Supplement: Translation

# Translation: Catheter Ablation of Focal Atrial Tachycardia with Early Activation Close to the His-Bundle from the Non Coronary Aortic Cusp

**Authors:** Muhieddine Chokr, Lucas G. de Moura, Italo Bruno dos Santos Sousa, Cristiano Faria Pisani, Carina Abigail Hardy, Sissy Lara de Melo, Arnobio Dias da Ponte, Ieda Prata Costa, Ronaldo Vasconcelos Tavora, Luciana Sacilotto, Tan Chen Wu, Francisco Carlos da Costa Darrieux, Denise Tessariol Hachul, Vera Aiello, Mauricio Scanavacca

**Affiliations:** 1 Brazil Universidade de São Paulo Faculdade de Medicina Hospital das Clinicas Instituto do Coração, São Paulo, SP – Brazil; 2 Brazil Antonio Prudente Hospital, Fortaleza, CE – Brazil

**Keywords:** Arrhythmias, Cardiac, Tachycardia, Atrial, Catheter, Ablation/methods, Bundle of His, Electrophysiologic,Techniques/methods, Electrocardiography/methods

## Abstract

**Background:**

Atrial tachycardia (AT) ablation with earliest activation site close to the His-Bundle is a challenge due to the risk of complete AV block by its proximity to His-Purkinje system (HPS). An alternative to minimize this risk is to position the catheter on the non-coronary cusp (NCC), which is anatomically contiguous to the para-Hisian region.

**Objectives:**

The aim of this study was to perform a literature review and evaluate the electrophysiological characteristics, safety, and success rate of catheter-based radiofrequency (RF) delivery in the NCC for the treatment of para-Hisian AT in a case series.

**Methods:**

This study performed a retrospective evaluation of ten patients (Age: 36±10 y-o) who had been referred for SVT ablation and presented a diagnosis of para-Hisian focal AT confirmed by classical electrophysiological maneuvers. For statistical analysis, a p-value of <0.05 was considered statistically significant.

**Results:**

The earliest atrial activation at the His position was 28±12ms from the P wave and at the NCC was 3±2ms earlier than His position, without evidence of His potential in all patients. RF was applied on the NCC (4-mm-tip catheter; 30W, 55ºC), and the tachycardia was interrupted in 5±3s with no increase in the PR interval or evidence of junctional rhythm. Electrophysiological tests did not reinduce tachycardia in 9/10 of patients. There were no complications in all procedures. During the 30 ± 12 months follow-up, no patient presented tachycardia recurrence.

**Conclusion:**

The percutaneous treatment of para-Hisian AT through the NCC is an effective and safe strategy, which represents an interesting option for the treatment of this complex arrhythmia. (Arq Bras Cardiol. 2021; 116(1):119-126)

## Introduction

Focal atrial tachycardias (AT) usually originate from certain structures comprised of atrial tissue, such as the *crista terminalis* , pulmonary veins, atrial appendages, and coronary sinus ostium. Radiofrequency (RF) catheter ablation has been established as the method of choice for the treatment of such arrhythmias. Although *foci* originating in the para-Hisian region are rare, they are a therapeutic challenge due to close proximity to the His-Purkinje system (HPS). As one attempts ablation via the right atrium, risk of affecting the AV node and HPS, thus causing atrioventricular (AV) block, would rise. However, the use of the retroaortic access to explore the non-coronary cusp (NCC), which is anatomically adjacent to the aforementioned region, is a well-known alternative strategy.^1^Experience with the efficacy and safety of this type of ablation remains limited. This study reports on a case series of para-Hisian atrial tachycardia that were mapped and ablated by the NCC. Electrophysiological characteristics and results with this approach were analyzed. Additionally, anatomy of the region and procedural strategies are discussed.

## Method

Records of 10 patients (8 women and 2 men; mean age: 36±10 years), from two Brazilian institutions (Heart Institute/InCor, University of São Paulo Medical School; Antonio Prudente Hospital, Fortaleza), subjected to catheter ablation between January 2014 and March 2017, were analyzed. Antiarrhythmic drugs were discontinued for at least five half-lives before the procedure. They were evaluated by physical examination, chest X-ray, and echocardiogram, and none of them showed structural heart disease.

The patients underwent electrophysiological study after 8-hours of fasting, under continuous monitoring and with a sedation level controlled by an anesthesiologist. Triple puncture was performed in the femoral vein, and standard catheters (3) were introduced in the coronary sinus (decapolar; 6F), His bundle region (quadrupole, 7F), and base of the right ventricle (quadripolar; 7F).

Programmed atrial stimulation or atrial burst was made with an EP-recording system (EP tracer; Netherlands) to induce tachycardia in two patients; spontaneous onset of tachycardia was observed in one patient; and isoproterenol (10-20mcg; IV infusion) was necessary in seven patients. In one case, an electroanatomic mapping system (Carto 3; Biosense) was available.

Diagnosis of AT was confirmed by using the following electrophysiological observations and maneuvers: changes in the A-A interval before changes in the V-V interval, ventricular entrainment during tachycardia with V-A-A-V-type response, or even changes in the V-A interval during tachycardia (absence of the V-A linking). In all cases, atrial activation with less than 50% of the tachycardia cycle length was observed, indicating a focal pattern of activation.

When the earliest atrial activation was in the right atrial septum and was followed by detectable His potential on the same site, the AT was defined as para-Hisian. Finally, the femoral artery was punctured in order to allow retrograde aortic valve region mapping in detail.

A 4-mm-tip therapeutic catheter was used for radiofrequency (RF) delivery (30W/55ºC during 60 seconds), taking the right and left oblique fluoroscopic incidences as references for anatomical location ( Figure 1 ). In one patient, an electroanatomic mapping system was used ( Figure 2 ). In all cases, the earliest activation site was identified by the NCC, regarding the onset of the peripheral P wave, similar to that detected by the catheter placed in the right interatrial septum, but with the advantageous absence of His bundle potential in the former ( Figure 3 ). Procedural success was defined as the termination of tachycardia during RF application, and non-induction of tachycardia after multiple attempts to induce it with atrial burst or after isoproterenol infusion.

**Figure 1:** The positioning of ablation catheter on the NCC in right and left oblique projection.

**Figure 2:** – Electroanatomic mapping showing RF application points on the NCC. Note the intimate relationship of the non-coronary cusp with the para-Hisian region.

**Figure 3:** – Sequentially from top to bottom: peripheral derivations, coronary sinus from proximal to distal electrodes, and recording catheters close to the aortic valve and His bundle region. Similar earliest local atrial activation time is observed in relation to the onset of the P wave from the septal para-hissian region and NCC, but with no His potential seen in the later.

### Statistical Analysis

Continuous data are given as mean ± standard deviation (SD) if normally distributed and as median plus interquartile range if not. Otherwise, counts and percentages (%) will be used for categorical data. The Shapiro-Wilk test was used to determine the normality of distribution. The Mann-Whitney U test was employed to compare differences between groups for non-parametric continuous values. Finally, the Fisher’s exact test was applied to assess the categorical data in a 2x2 contingency table. For all tests, a *p* -value of <0.05 was considered statistically significant (2-sided). SPSS software version 19.0 (SPSS, Inc, Chicago, Illinois) was used for statistical analysis.

## Results

The Clinical and electrophysiological characteristics of patients can be seen in Tables 1 and 2. All continuous variables, except for P-wave duration during sinus rhythm and tachycardia, displayed normal distribution ( Table 2 ).

**Table 2:** – The electrophysiological characteristics of the evaluated patients

Variable	Value
Mean age (years)	36 ± 10*
Duration of the p wave during tachycardia (ms)	93 ± 17§
Duration of the p wave during sinus rhythm (ms)	112 ± 20§
Ablation success rate	9/10 (90%)
Mean tachycardia cycle length (ms)	362±43*
Earliest atrial activation recorded on His catheter (ms)	28±12*
Earliest activation site to his-bundle region (ms)	3±2*
Mean fluoroscopy time (minutes)	14 (10 – 18)
Time from ablation start to tachycardia interruption (seconds)	5 (2 – 8)
Use of 3-D mapping system	1/10
Coronary angiography	0/10
Major complications	0/10

*Data is presented as mean + standard deviation (SD). §Value is displayed as median + interquartile range (IQR).

None of the patients had previously undergone catheter ablation. The mean atrial tachycardia cycle length was 362±43 ms. Earliest atrial activation recorded on His catheter was 28±12 ms in relation to the peripheral P wave. The atrial local activation time recorded by the catheter on the NCC cusp was 3±2 ms earlier compared to the His catheter.

In all cases, the initial ablation site was the NCC, and was successful in 9 of 10 cases. The remaining case required mapping and attempt of ablation in the para-Hisian region with low power (20w), which was unsuccessful as well.

The mean time to atrial tachycardia interruption after RF delivery was 5 seconds. Junctional rhythm and an increase in the PR interval were not observed during application of RF in all cases. All procedures were well tolerated and without complications.

Over a follow-up of 30 ± 12 months, no patient presented a recurrence of atrial tachycardia and remained asymptomatic in clinical evaluation and with dynamic continuous ECG Holter monitoring.

Regarding the surface electrocardiogram, it was observed that the morphology of the P wave demonstrated a biphasic or triphasic pattern in 6 of 10 patients in inferior leads and were significantly shorter in duration compared to the sinus rhythm in all cases 93 ± 17 vs. 112 ± 20 ms (p<0.05). ( Figure 4 ).

**Figure 4:** – P-wave morphology of all cases. The morphology of the P wave, demonstrating biphasic or triphasic patterns in 6 of 10 patients in inferior leads.

## Discussion

### Morphological relations

The aorta occupies a central position at the base of the heart, deeply wedged between the right and left atrioventricular junctions. The spatial relations of the sinuses of Valsalva thus demonstrate proximity with the atrial walls and the adipose tissue interposing between them at the base of the heart. Considering the semilunar pattern of attachment of the aortic leaflets, it is evident that the topographical relations vary according to the deepness inside the sinus. Figure 5A and 5B shows the anatomical relations of the non-coronary aortic sinus relative to the right atrial structures. In particular, the deepest portion of the non-coronary (also called the non-adjacent) aortic sinus is very closely related to the atrioventricular component of the cardiac septum. Figure 5C shows the sinus wall and the landmarks of the junctional area and atrioventricular node

**Figure 5:** – An intimate relationship between the NCC and the His bundle region can be observed. A) Oblique view of a short axis section at the base of the heart, showing the non-coronary sinus of Valsalva (NC) and the landmarks of the triangle of Koch (dotted lines) and membranous septum (star). B) Short axis section at the base of the heart showing the spatial relations of the aortic sinuses and the adipose tissue present between the aorta and the atrial walls. C) Longitudinal section of the aortic root showing the short distance between the deep portion of the non-coronary sinus of Valsalva (NCC) and the area corresponding to the apex of the triangle of Koch, located antero-superiorly to the coronary sinus orifice (double-headed arrow).

Thus, the NCC becomes an alternative target within a therapeutic strategy in which failure in intervention occurs when ablation is attempted on both sides of the atrial septum or risk of atrioventricular block occurs as a result of the electrogram record of His bundle close to the ablation target.^2^

From the embryological point of view, neural crest cells contribute to form the aortopulmonary septum, endocardial cushion in the outflow tract, and isolation of the His bundle from the surrounding myocardium. Remnants of these cells on the perinodal region can justify the substrate, which gives rise to maintains arrhythmia.^3^The NCC originates from atrial myocardium, while the right and left coronary cusp originate from ventricular myocardium. This fact explains the frequency of atrial arrhythmias in the NCC and ventricular arrhythmias in the right and left cusps.^3^

Prevalence of tachycardias originating in the perinodal region is about 7-10% in different series with several series, and case reports have shown that para-Hisian tachycardias can be adequately treated with a low complication rate.^4^

Approach of these tachycardias through the NCC reduces the risk of damage to the conduction system, providing a greater stability to the catheter during RF application, as well as good contact with the tissue. Targeting the right atrial extensions at the NCC, farther from the His Purkinje system, which is situated in the endocardium, is the likely explanation for ablation being effective at this site.^5^

With regard to complications, RF application can cause damage to the heart valves, although this complication has not been reported up to the limits of power (30w) and temperature (55°C) in several series.^6^Coronary angiography was not routinely performed before applying RF because, in our practice, the presence of a local electrogram with atrium greater than the ventricle (A/V ratio >1), anatomically close to a catheter used as a reference in the right atrium, parallel to the conduction system, marks a safe ablation site. Regarding the mapping technique, a ratio greater than or equal to 1 between the amplitudes of the atrial and ventricular electrograms was observed in all patients in the ablation target. This electrophysiological feature is of great value because inversion of this A/V relationship suggests that the limit of the NCC is being crossed and the catheter is then supported over the right cusp. This leads to a greater risk of injury to the conduction system, thus serving as an aid and anatomical reference when only fluoroscopy is used.^7^

Electroanatomical mapping (EAM) was used in only one case. The reason for this is that most of our patients came from the public health system, where the above procedure is not available. However, in our sample, as described by Toniolo et al.,^8^it was possible to achieve high success rates despite not using it.

On the other hand, there are situations in which EAM is essential. Bitaraes et al. recently published a case of a pregnant woman with a focal AT refractory to pharmacological treatment, in which the Catheter ablation was successfully performed by the non-coronary aortic cusp with zero fluoroscopy, using only EAM.^9^

Our findings disagree with those of Ouyang et al.,^10^who observed -/+ P wave in the V1 lead associated with P+ in D1 and AVL suggested NCC origin. According to this author, the relationship between the presence of the -/+ P wave, with its most prominent portion being positive, and origin in the left atrium is a relevant fact. Recently, Madaffari et al.^11^published data of P-wave morphology, where a characteristic narrow and biphasic (-/+) or triphasic (+/-/+) P wave in the inferior and precordial leads reliably identifies the group of AT arising from the para-Hisian region. The present study found that the morphology of the P wave, demonstrating a biphasic or triphasic pattern in 6 of 10 patients in inferior leads and a significantly shorter P wave when compared to the sinus rhythm, was variable at the precordial leads.

In our study, an unsuccessful attempt to ablate tachycardia by the NCC occurred in only one of the ten cases, which was ineffective from the right atrium as well. It was assumed that a more aggressive strategy on the right side of the septum could have resulted in both damage to the conduction system and atrioventricular block, justifying the low power output tested (20w). Tachycardia stopped during the applications, but it could be induced again during the infusion of isoproterenol. A deeper target in the septal region could explain the difficulty in eliminating the substrate. Another limitation is that the operator did not explore the left septal region in this case. An irrigated catheter was also not used because, in our opinion, the aortic root is a high blood flow region and unless power delivery was repeatedly limited by high temperature cutoffs, irrigation should not make a significant difference. In the clinical follow-up, the patient was asymptomatic, under the use of betablockers. Thus, no new ablation attempt was performed.

Recently Lyan et al.^12^evaluated different strategies for catheter ablation of focal atrial tachycardia originating near the His bundle region in 68 patients and found that the acute success rate of para-Hisian AT ablation at the NCC was higher than that of ablation at the LA septum and at the RA septum (p<0.05). For this reason, they sustain that NCC must be the first and preferred approach to these tachycardias regardless of the local activation time, which rins in line with findings from Bohora et al.^13^By contrast, Madaffari et al.^11^sustain that NCC is only one of three possible approaches to achieve success, and the choice should be based on the local activation time.

Our findings are in agreement with Lyan et al.^12^and Bohora et al.^13^as the NCC approach should be the first choice to perform ablation in this scenario with high success rates.

## Conclusion

This study confirms previous observations that the mapping and ablation of focal atrial tachycardia with early activation close to the His-Bundle from the non-coronary aortic cusp (NCC) is an effective and apparently safe procedure. It can therefore be concluded that retroaortic exploration should be mandatory in such cases. A surface electrocardiogram can suggest the suitable target near the His-Bundle region but not in all cases. The knowledge of the relations of the NCC with the conduction system is crucial in the ablation of these tachycardias. These findings should be considered in the therapeutic strategy of this complex arrhythmia.

**Table 1:** – Clinical characteristics of the evaluated patients

Patient	Age	Sex	Structural heart disease	Duration of symptoms (months)	Ineffective AADs	Past Failed Ablation
1	38	F	None	≤12	0	NO
2	49	M	None	12-24	2	No
3	22	F	None	12-24	1	No
4	28	F	None	12-24	0	No
5	31	F	None	≥48	3	No
6	33	F	None	≥48	1	No
7	46	F	None	≤12	1	No
8	58	F	None	12-24	2	No
9	25	M	None	≤12	0	No
10	30	F	None	≤12	1	No

*AADs: antiarrhythmic drugs.*
